# Evidence That a Peptide-Drug/p53 Gene Complex Promotes Cognate Gene Expression and Inhibits the Viability of Glioblastoma Cells

**DOI:** 10.3390/pharmaceutics16060781

**Published:** 2024-06-08

**Authors:** Ana Neves, Tânia Albuquerque, Rúben Faria, Cecília R. A. Santos, Eric Vivès, Prisca Boisguérin, Diana Carneiro, Daniel F. Bruno, Maria D. Pavlaki, Susana Loureiro, Ângela Sousa, Diana Costa

**Affiliations:** 1 CICS-UBI—Health Sciences Research Centre, University of Beira Interior, 6201-001 Covilhã, Portugal; a34643@ubi.pt (A.N.); tania.albuquerque@ubi.pt (T.A.); ruben.faria@ubi.pt (R.F.); csantos@fcsaude.ubi.pt (C.R.A.S.); angela@fcsaude.ubi.pt (Â.S.); 2 PhyMedExp, INSERM, CNRS, University of Montpellier, 34295 Montpellier, France; eric.vives@umontpellier.fr (E.V.); prisca.boisguerin@inserm.fr (P.B.); 3 CESAM—Centre for Environmental and Marine Studies, Department of Biology, University of Aveiro, 3810-193 Aveiro, Portugal; dianaisabel@ua.pt (D.C.); danielbruno@ua.pt (D.F.B.); maria.pavlaki@ua.pt (M.D.P.); sloureiro@ua.pt (S.L.)

**Keywords:** apoptosis, caspases, cell-penetrating peptides, co-delivery, glioblastoma therapy, p53

## Abstract

Glioblastoma multiform (GBM) is considered the deadliest brain cancer. Conventional therapies are followed by poor patient survival outcomes, so novel and more efficacious therapeutic strategies are imperative to tackle this scourge. Gene therapy has emerged as an exciting and innovative tool in cancer therapy. Its combination with chemotherapy has significantly improved therapeutic outcomes. In line with this, our team has developed temozolomide–transferrin (Tf) peptide (WRAP5)/p53 gene nanometric complexes that were revealed to be biocompatible with non-cancerous cells and in a zebrafish model and were able to efficiently target and internalize into SNB19 and U373 glioma cell lines. The transfection of these cells, mediated by the formulated peptide-drug/gene complexes, resulted in p53 expression. The combined action of the anticancer drug with p53 supplementation in cancer cells enhances cytotoxicity, which was correlated to apoptosis activation through quantification of caspase-3 activity. In addition, increased caspase-9 levels revealed that the intrinsic or mitochondrial pathway of apoptosis was implicated. This assumption was further evidenced by the presence, in glioma cells, of Bax protein overexpression—a core regulator of this apoptotic pathway. Our findings demonstrated the great potential of peptide TMZ/p53 co-delivery complexes for cellular transfection, p53 expression, and apoptosis induction, holding promising therapeutic value toward glioblastoma.

## 1. Introduction

Glioblastoma multiform (GBM) is the most common malignant adult brain cancer, characterized by a poor prognosis (approximately 15 months after diagnosis) [1,2,3]. According to the World Health Organization (WHO) classification, it is considered a grade IV glioma. Besides significant advances in diagnosis and therapeutics, GBM has remained a deadly disease without statistically enhanced patient survival rates for more than two decades [4]. Currently, the most common treatment applied to GBM includes surgical resection of the tumor, followed by radiotherapy and/or chemotherapy, mainly based on the use of temozolomide (TMZ) [3,5,6,7,8]. However, these therapeutic strategies fail to increase survival outcomes due to the unique molecular characteristics of GBM, namely the presence of a stem-like cell population (glioma stem cells) that greatly contributes to its resistance to radiotherapy and chemotherapy [9,10,11]. Additionally, the presence of the blood–brain barrier (BBB) limits the passage and penetration of drugs, preventing them from reaching the tumor [12,13]. Therefore, there is an urgent need for novel and innovative therapies. In the presented work, the strategy involves the delivery of complexes locally and directly, where the tumor has been removed, by intracranial injection. This way, we can overcome the typical problems associated with BBB penetration and bloodstream removal. Several works following this approach to treat gliomas have already been published and some of them suggested that this route of administration can lead to longer retention time at the tumor location and higher therapeutic effect [12,14,15].

Gene therapy has long been recognized as a promising technology for many severe diseases, such as cancer, due to its potential therapeutic value [16,17,18]. Gene therapy focused on re-establishing p53 function has been extensively explored for cancer treatment [19,20,21]. p53 is a tumor suppressor protein involved in maintaining genome integrity, as it can induce cell cycle arrest at various stages or induce apoptosis upon cellular stress. Mutations in the p53 gene are very frequent in human cancers, accounting for more than 50%, including brain, colon, lung, breast, or stomach cancers [22,23]. Dysregulated p53 pathway components have been linked to several processes in GBM, such as cell invasion, proliferation, evasion of apoptosis, or cell stemness. For instance, the p53-ARF-MDM2 pathway is dysregulated in a quite high percentage of GBM cell lines (94%) and patients (84%) [24]. 

Beyond gene therapy, combination therapy for cancer has gained notoriety over recent years [25,26,27]. The co-delivery of different therapeutics contributes, mainly, to tumor inhibition and reduced dosage of anticancer agents due to the synergistic effect of their combined action. In this sense, the dual delivery of an anticancer drug and the p53 gene has been strategically explored to achieve high levels of cancer cell death efficiency [25,27,28,29]. For example, p53 upregulation may increase the sensibility of these cell lines to TMZ action by suppressing O-6-methylguanine-DNA methyltransferase (MGMT) expression, a DNA enzyme that reverts the process of TMZ-induced DNA damage [30,31,32,33,34]. Furthermore, the p53 tumor suppressor protein has been shown to regulate the expression of some DNA methyltransferases (DNMTs) responsible for methylating DNA groups [35,36]. p53 also induces apoptotic TMZ-independent apoptosis and leads to cell cycle G1 arrest and participates in the activation of several transcriptional genes involved in DNA repair pathways and senescence [37]. Therefore, we hypothesized that supplementation of p53 may be an effective strategy to overcome TMZ resistance treatment in these cell lines.

To accomplish dual delivery, the conception of a nano-delivery platform suitable to complex, protect, transport, and release both drugs and genes is imperative. In this context, the design/development of non-viral delivery systems to promote cellular uptake and intracellular targeted payload delivery has been considered a convenient tool to increase therapeutic efficacy in cancer gene therapy [38,39,40]. Cell-penetrating peptides (CPPs) are short peptides composed of less than 30 amino acids that can carry biomolecules and easily translocate through the cell membrane—a property related to the amino acid sequence [41,42,43]. Although the mechanism of CPP penetration is not fully understood, it seems to be influenced by factors such as the cell type, membrane composition, properties of the cargo, or peptide/cargo ratio [44,45,46]. Moreover, CPPs can be engineered to target specific organelles or cells and, therefore, CPP-based delivery systems are promising platforms to mediate cancer therapy, as they can enhance cell targeting, improve cell uptake, and reduce toxicity. Following this, clinical trials evidenced the potential of CPP-based vehicles for cancer treatment [47,48,49]. Applied to GBM, CPPs were revealed to be of extreme utility in overcoming the main obstacles to successful therapy, such as poor BBB penetration, inefficient tumor targeting, or low TMZ cytotoxicity [50,51]. In line with these findings, in previous work, our research group reported on the development of a CPP bearing TMZ along with GBM targeting affinity, designated as TMZ/Tf-WRAP5, and on the formulation of TMZ/Tf-WRAP5/p53 delivery systems to target U87 cells and promote dual delivery [43]. In the present report, information regarding the biocompatibility of these systems in non-cancerous cell lines and a zebrafish embryo model is described. Zebrafish appear as a suitable animal model, especially in the embryonic development phase of the life cycle, to study delivery systems in vivo biocompatibility. Several studies have used *Danio rerio* to perform those experiments successfully [52,53,54,55]. Additionally, the systems’ targeting ability was investigated in SNB19 and U373 glioma cells, as well as their capacity for p53 expression. From this, the effect of the novel nano-platform on cancer cell viability inhibition was evaluated, and its role in apoptosis induction was addressed. The present study reveals that the developed nano-complexes provide a powerful, safe, and efficient co-delivery tool for potential in situ glioblastoma therapy strategy by intracranial injection. The strategy involved direct delivery of the complexes, after the standard procedure of tumor resection, to promote a therapeutic effect, an already tested approach by other researchers [12,14,15]. 

## 2. Materials and Methods

### 2.1. Materials

WRAP5 and Tf-WRAP5 syntheses were performed on the LibertyBlue™ Microwave Peptide synthesizer (CEM Corporation, Matthews, NC, USA) with an additional Discover™ module (CEM Corporation, NC, USA) combining microwave energy at 2450 MHz with the Fmoc/*tert*-butyl (*t*Bu) strategy. The peptides were supplied as lyophilized powders and kept at 4 °C until use. The plasmid pcDNA3-FLAG-p53, 6.59 kbp (Addgene plasmid 10 838, Cambridge, Massachusetts, USA), was produced and purified by following a procedure created and optimized by our research group, fully described elsewhere [56]. The drug temozolomide (TMZ) was purchased from Frilabo (Lisbon, Portugal), 3-(4,5-dimethylthiazol-2-yl)-2,5-diphenyltetrazolium bromide (MTT) and fluorescein isothiocyanate (FITC), isomer 1, were obtained from Sigma Aldrich Chemicals (St. Louis, Missouri, USA). The dye DAPI was acquired from Invitrogen (Carlsbad, CA, USA). Dulbecco’s modified Eagle’s medium (DMEM)/Ham’s F-12 Nutrient Mixture (DMEM/F-12) with L-glutamine was purchased from Sigma-Aldrich (St. Louis, Missouri, USA), and high-glucose DMEM with stable L-glutamine from Biowest (Nuaillé, France). Penicillin–streptomycin–amphotericin B solution was obtained from the American Type Culture Collection (ATCC, Manassas, VA, USA). A p53 ELISA kit was purchased from Roche (Roche Applied Science, Penzberg, Germany). An ApoAlertTM Caspase-3 Colorimetric assay kit and Caspase-Glow^®^ 9 Assay were acquired from Promega (Madison, WI, USA). A Human BAX ELISA Kit (No-EH0669) was purchased from Universal Biologicals (Cambridge, UK). Ultrapure grade water, purified with a Milli-Q system from Millipore (Billerica, MA, USA), was used to prepare all the solutions. A human normal astrocyte cell line, HA1800, and astrocytes type I (CTX-TNA2) are from ATCC. Human GBM U373MG cells (mutant p53) were a gift from Dr. J. Costello (University of California, San Francisco, USA), and SNB19 cells (mutant p53) were obtained from the German Collection of Microorganisms and Cell Cultures. U-87 human cells, a cell line isolated from malignant glioma from a male patient, likely with glioblastoma, were supplied by the European Collection of Authenticated Cell Cultures (ECACC, Salisbury, UK).

### 2.2. Methods

#### 2.2.1. Formation of pDNA Complexes

The preparation of TMZ-loaded peptides was already described in a previous publication from our team [43]. After synthesis and TMZ loading, peptides were suspended in ultrapure water and kept at −20 °C. The solution concentration was acquired by measuring the sample absorbance at 280 nm in a Nano-Photometer™ (Implen, Inc., Westlake Village, CA, USA). The pDNA complexes were prepared at an N/P of 1 (nitrogen to phosphate groups (N/P) ratio) by pipetting 50 µL of peptide solutions into 150 µL of pDNA solution (comprising 1 µg in 10 mM Tris-EDTA pH 7.0 buffer) dropwise and vortexing for 60 s. The formed complexes were left for equilibration for 25 min at room temperature. Afterward, the mixture was centrifugated at 13,500× *g* for 20 min at 4 °C. The pellet, containing the nano-complexes, was used in later assays.

#### 2.2.2. Characterization of the pDNA Complexes

The morphology of the peptide/pDNA complexes developed at N/P ratios of 0.5 and 1 was determined by scanning electron microscopy (SEM). The microscopy images have been reported in a previous publication [43].

The properties of peptide/pDNA complexes, such as the mean size, polydispersity index (PdI), and zeta potential, were determined by dynamic light scattering (DLS). Furthermore, the pDNA complexation capacity (CC) was determined by using the Nano-Photometer™ device (Implen, Inc., Westlake Village, CA, USA) to measure the concentration of pDNA and by using Equation (1). Details of the experiments are fully described elsewhere [43].

CC (%) = [(Initial amount of pDNA) − (non-bound pDNA)]/Initial amount of pDNA × 100(1)

#### 2.2.3. Cell Culture

HA1800, CTX-TNA2, U87, SNB19, and U373 cells were cultivated in 75 cm^3^ t-flasks with high-glucose DMEM with stable L-glutamine medium, pH 7.4. This medium was supplemented with 10% heat-inactivated FBS and a 0.1% (*v*/*v*) mixture of penicillin (100 µg/mL) and streptomycin (100 µg/mL). The cellular growth was promoted at 37 °C and 5% of CO_2_ until ~80% confluence was attained. Cells were sub-cultivated every 3 days to maintain their exponential growth and normal metabolism.

#### 2.2.4. Cytotoxicity Assessment

The cytotoxicity profile of the nano-complexes was assessed on human normal astrocytes, HA1800, CTX-TNA2 (astrocytes type I), SNB19, and U373 cells using the 3-[4,5-dimethyl-thiazol-2-yl]-2,5-diphenyltetrazolium bromide (MTT) assay. Cells were plated in 96-well plates (10^4^ cells per well) and grown at 37 °C and 5% CO_2_. At least 12 h before transfection, the starvation method was applied. On the day of the transfection, 0.1 µg of pDNA was pipetted into each well and left for 4 h. The previous medium was changed to a new medium supplemented with FBS and a mixture of antibiotics to end the transfection process. The percentage cellular viability in relation to control wells was determined by [A]_Sample_/[A]_Control_ × 100. A Benchmark Microplate Reader (BioRad, Vienna, Austria) was used to measure the absorbance at 570 nm. The absorbance of DMSO was subtracted from all measured samples. The positive control refers to non-transfected cells. Moreover, cellular transfection was mediated by naked pDNA or TMZ, and these data were also taken as controls.

#### 2.2.5. Animal Care

Embryo–larval zebrafish were used to carry out the in vivo experiments. *Danio rerio* (zebrafish, wild-type AB) eggs were obtained from the DGAV (provisional operating permit for culture and use of zebrafish) accredited facility at the Biology Department, University of Aveiro (Portugal). Zebrafish were kept growing in a ZebTEC recirculating activated-charcoal-filtered water system (Tecniplast) complemented with Instant Ocean Synthetic Sea Salt (Spectrum Brands, Middleton, WIS, USA) (conductivity 750 ± 50 µS/cm; pH 7.5 ± 0.3, dissolved oxygen ≥ 95% saturation, and salinity 0.34 mg/L) with a temperature of 26 ± 1 °C and under a photoperiod cycle of 12:12 h (light:dark). Fish were fed twice a day with an artificial diet of Gemma Micro 500 (Skretting^®^, Burgos, Spain). 

#### 2.2.6. Fish Embryo Toxicity (FET) Tests 

The fish embryo toxicity (FET) test was performed to assess the toxicity of the systems according to the OECD 236 guideline [57]. The day before the test, healthy and sexually mature males and females (2:1) were separated by a physical barrier in an aquarium. On the day of the test, the physical barrier was removed, and males and females were allowed to mate for 1 h. After that, fertilized eggs were collected and checked under a stereomicroscope (Stereoscopic Zoom Microscope—SMZ 1500, Nikon, Tokyo, Japan). Eggs with any malformations or non-fertilized eggs were discarded following the OECD 236 guideline. Three hours post-fertilization (hpf), zebrafish embryos were transferred to 24-well plates (1 egg per well in 2 mL of exposure media) and exposed to different concentrations of the various systems. Ten embryos and four internal controls were used per plate and three plates per concentration. A negative control (only fish system water (FSW), for validation purposes), a solvent control (DMSO (100 µ/L of FSW); whenever TMZ was tested), and a positive control (4 mg/L 3,4-dichloroaniline (3.4-DCA)) were always considered. TMZ drug was, in agreement with the manufacturer’s instructions, dissolved and prepared in DMSO solvent at a concentration of 1 g/L. According to the OECD recommendations regarding the maximum solvent volume, in each well plate the maximum TMZ concentration tested was 1 mg/L (5.2 µM). The development of the zebrafish eggs, embryos, and larvae was followed and observed daily under the Stereoscopic Zoom Microscope—SMZ 1500, Niko—during 96 h of exposure period to record the hatching success, malformations, and survival. 

FSW was used to prepare all the tested solutions. Water quality parameters such as pH, dissolved oxygen, conductivity, and temperature were measured before and after the experiments, as indicated by the OECD guideline 236.

#### 2.2.7. Live Cell Imaging Assay

##### FITC Plasmid Labeling

The labeling of plasmid DNA (pDNA) with FITC was performed by combining 2 µg of pDNA with 2 μL of FITC (50 mg/100 µL in anhydrous dimethyl sulfoxide (DMSO)) and 81 µL of labeling buffer (0.1 M sodium tetraborate, pH 8.5). Solutions were stirred at room temperature for 4 h. To end the reaction, 2.5 volumes of ethanol 100% and one volume of 3 M NaCl were pipetted into the mixture and incubated at −20 °C for 30 min. Next, samples were centrifuged at 4 °C (10,000× *g*, 30 min). The pDNA-FITC pellet was washed with 300 µL of ethanol 75% and centrifuged again at 10,000× *g* for 10 min until loss of orange tone. Then, pellets were resuspended in ultrapure grade water to form the complexes. The experiment was performed in dark conditions to protect the samples from light interference.

##### Cellular Internalization

The cellular internalization of the developed nano-complexes was investigated by considering a live cell experiment and using a Zeiss LSM 710 confocal laser fluorescence microscope (CLSM) (Carl Zeiss SMT, Inc., Oberkochen, Germany). First, SNB19 and U373 cells were plated (25,000 cells/well) in an 8-well µ-slide (Ibidi, Martinsried, Germany), then, approximately 12 h before transfection, the medium was changed to FBS and antibiotic-free medium (cell starvation). On the day of the confocal microscopy experiment, 0.25 µg of pDNA-FITC was pipetted into each well. Two hours post-transfection and immediately before image acquisition, the nucleus was stained with 1 µM DAPI for 10 min. Images were acquired with ZEN microscopy software 3.7 after 2 and 4 h of transfection. During the acquisition, cells remained at 37 °C. For the acquisition of cell images, the respective laser and filters of each dye were considered, DAPI (Diode 405-30 laser unit, λ = 405 nm) and FITC (Argon/2 laser unit, λ = 488 nm). The Zeiss Zen 3.7 software was used to process/analyze the obtained images.

#### 2.2.8. Protein Quantification

Analysis of p53 expression levels on cells was carried out with the p53 pan ELISA kit (Roche Applied Science, Penzberg, Germany) according to the procedure described by the manufacturer. This kit is based on the principle of sandwich ELISA in which the protein of the samples binds to an immobilized capture antibody and then to a detection biotin-labeled antibody that interacts with an UltraAvidin–horseradish peroxidase (HRPO) conjugate. This conjugate reacts with a tetramethylbenzidine substrate to produce a colored product that is quantified spectrophotometrically. Initially, glioblastoma cells were plated in 12-well plates (10^5^ cells per well) and, at least 12 h before transfection, the starvation method was applied. On the transfection day, 1 µg of pDNA was pipetted into each well and left for 4 h. Transfection was stopped by changing the previous medium with a new medium supplemented with FBS and a mixture of antibiotics. Forty-eight hours after transfection, the medium was taken out and cells were washed three times with phosphate buffer solution (PBS, 1×). Centrifugation was used to recover the cell pellet and cell lysis was performed using a detergent composed of 1% Triton X-100, 0.1% SDS in PBS, pH 7.4, and protease inhibitor cocktail. After the sample incubation with antibodies, p53 was spectrophotometrically determined at 450 nm using a spectrophotometer (Shimadzu UV–vis 1700 from Shimadzu, Duisburg, Germany). Non-transfected cells were considered as the negative control. Also, cells transfected with naked pDNA or TMZ were taken as controls.

#### 2.2.9. Caspase-3 and Caspase-9 Quantification

The transfection and recovery protocols were similar to those described above for p53 protein quantification. Caspase-3 or caspase-9 were determined using the ApoAlertTM Caspase-3 and Caspase-Glow^®^ 9 Assay (Promega, Madison, WI, USA), respectively. The quantifications were carried out according to the procedure described by the manufacturer, namely the incubation of the cells with staurosporine (1 µM) during the same transfection time; this was considered the positive control. Caspase-3 enzyme activity was evaluated by measuring spectrophotometrically, at 405 nm, the chromophore p-nitroaniline (pNA) from the labeled substrate DEVD-pNA cleavage. The activity of caspase-9 was evaluated in turn through the quantification of a homogeneous luminescent signal resulted from caspase cleavage of the luminogenic caspase-9 substrate.

#### 2.2.10. Bax Quantification

Bax protein levels were determined quantitatively using the Human BAX ELISA Kit (No-EH0669) according to the manufacturer’s indications. In summary, the lysis of glioma cells proceeded according to ELISA kit instructions. Bax protein in cell lysates binds to the primary antibody and was detected by horseradish peroxidase–secondary antibody conjugate. The amount of Bax was determined by reading its absorbance at 450 nm in a microplate reader.

#### 2.2.11. Statistical Analysis

Student’s *t*-test was performed to check for statistically significant differences between solvent control (DMSO) and the negative FSW control in the TMZ drug FET experiment. No statistical differences were found. One-way or two-way analysis of variance (ANOVA) was considered in statistical analysis, with the addition of the Bonferroni test. 

Data are quantitatively presented as the mean ± standard deviation (SD) and were analyzed using GraphPad Prisma software, V9.0.0 (GraphPad Software Inc., New York, NY, USA). 

## 3. Results and Discussion

### 3.1. Characterization of Peptide-Based Complexes 

TMZ/WRAP5 and TMZ/Tf-WRAP5 were prepared according to an established protocol presented elsewhere [58]. The final product was derived from the covalent bonding of the amino groups (-NH_2_) of the N-terminal leucine and (-NH) groups of tryptophane residues, and the guanidinium group (-HN-C(NH)(NH2)) from arginine was derived from WRAP5 peptide with methyldiazonium ion (N≡N+) resulting from TMZ hydrolysis in water (please consult Scheme S1 of the Supplementary Materials (SM)). After that, the amount of TMZ loaded in the peptides was determined by HPLC, and TMZ-loading efficiency was around 60% and 66% for TMZ/WRAP5 and TMZ/Tf-WRAP5, respectively (Table 1). Peptide/pDNA complexes were formulated at several N/P ratios by both electrostatic and hydrophobic interactions, resulting in the creation of particles at the nano-scale, bearing a spherical shape [46,59]. The electrostatic interaction between the WRAP5 peptide and the plasmid is favored by arginine residues of WRAP5, while their tryptophan residues are supposed to interact by hydrophobic forces with the minor groove of pDNA [60]. After formulation, the set of peptide-based complexes was adequately characterized to determine their physicochemical characteristics, such as morphology, mean size, polydispersity index, and superficial charge. Additionally, the pDNA complexation behavior was monitored by agarose gel electrophoresis. For complexes prepared at an N/P ratio of 1, and as a function of system type, pDNA complexation capacities may reach 89–95%. This result indicates the effective ability displayed by WRAP5 to complex pDNA. Stability studies were also performed and demonstrated that the formed nano-vectors are stable and protect pDNA from degradation [43]. From these previous studies, we found that the nano-systems developed at an N/P ratio of 1 are the most adequate complexes to promote cellular uptake and payload delivery, as they exhibited a spherical shape and an apparently smooth surface, possessing sizes below 200 nm (allowing for a targeted internalization via receptor-mediated endocytosis), positive zeta potential, and higher pDNA complexation capacity (CC) (Table 1). We concluded that no advantage arises in considering the formation of peptide/pDNA systems at higher ratios [43]. Regarding the BBB, several studies showed positive results when crossing the barrier with nano-complexes ranging in sizes from 100–200 nm, even in vivo [61,62,63]. 

Furthermore, an incubation in 10% serum demonstrated the ability of complexes to protect the genetic material against serum degradation, as shown in the Supplementary Materials (Figure S1). A hemolysis test proved that the complexes have low toxicity to red blood cells (RBCs) with a maximum of hemolytic activity of 17.32 ± 1.72% for the complex bearing transferrin only (Figure S2). As a maximum of 10% could be considered safe for in vivo studies, the developed nano-complexes were demonstrated to be safe, with the exception of Tf-WRAP5/pDNA complexes [64]. At this stage, we should highlight that future in vivo studies will be carried out with TMZ/Tf-WRAP5/pDNA complexes, the most promising ones, that exhibited a hemolysis activity of 7.87 ± 0.23%. Cytokine expression comes from an immune response after antigen recognition by dendritic cells. Despite complexes proved to be hemocompatible it is important to evaluate if they can provoke an inflammatory response when possibly administered in vivo. In this way, the proinflammatory interleukin-6 (IL-6) and interleukin 1β (IL-1β) secretion after JAWS II cell exposure to nano-complexes was studied to investigate if the complexes trigger the production of these cytokines. The results are presented in Figure S3 of the Supplementary Materials. As can be observed, the transfection of JAWS II cells with the developed nano-complexes did not trigger a significant statistical increase in both IL-6 and IL-1β cytokine levels when compared with the basal level of control cells. Therefore, the peptide nano-complexes seem to be suitable for administration in vivo without activating the innate immune system. Our data suggest that the developed carriers are suitable even for intravenous administration and therapeutic gene release in vivo.

These findings instigated us to further explore the performance of peptide/pDNA nano-systems at an N/P ratio of 1, namely their targeting/delivery ability and, consequently, their role in apoptosis induction. Therefore, in the present report, across the following sections of discussion, we present in vivo and in vitro studies aiming to address various processes mediated by these nano-complexes, such as the biocompatibility, cellular uptake, p53 protein expression, inhibition of glioma cell viability, and apoptosis. SNB19 and U373 glioma cell lines, both p53 mutated, were chosen to perform the in vitro studies to understand the impact of p53 status on nano-complex performance and to compare the obtained data with the previous work, where U87 p53 wild-type cells were used [43]. Recent studies have suggested a relation between TMZ action resistance and p53 status [65,66,67,68]. 

### 3.2. The Peptide-Based Complexes Are Biocompatible to Non-Cancer Cells

**Figure 1:**
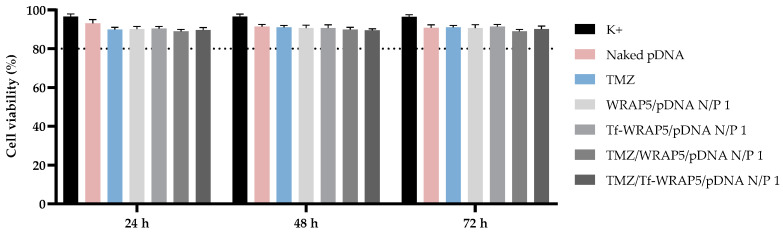
Cellular viability of HA1800 cells after 24 h, 48 h, and 72 h of transfection mediated by the different peptide/pDNA complexes developed at N/P ratio of 1 (using 1 µg pDNA). Cells not transfected were used as a positive control and cells treated with naked pDNA and TMZ drug were used as controls. Data were obtained from six independent measurements (mean ± SD, n = 6).

**Figure 2:**
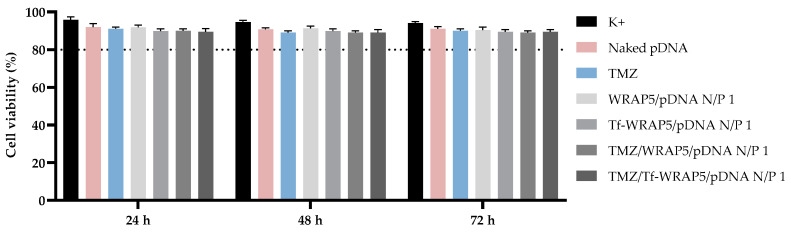
Cellular viability of CTX-TNA2 cells after 24 h, 48 h, and 72 h of transfection mediated by the different peptide/pDNA complexes developed at N/P ratio of 1 (using 1 µg pDNA). Cells not transfected were used as a positive control and cells treated with naked pDNA and TMZ drug were used as controls. Data were obtained from six independent measurements (mean ± SD, n = 6).

Testing the cytotoxicity of nanoparticles in vitro by the MTT assay is still one of the most applied methods to determine cellular viability and proliferation. Therefore, the cellular viability of human normal astrocytes, HA1800, and CTX-TNA2 (astrocytes type I) was assessed at 24 h, 48 h, and 72 h of transfection, mediated by the developed complexes, by MTT colorimetric assay. Figure 1 and Figure 2 display the cellular viability percentage achieved after transfection with the different peptide/pDNA complexes (N/P ratio of 1), highlighted in Table 1.

In HA1800 cells (Figure 1), for the three time points considered, a superior 80% value was achieved for all treatment groups. Pursuant to ISO 10993-5 [69], if an 80% or superior cell viability is obtained, then substances are considered non-cytotoxic. Therefore, these results have shown that none of the developed systems induced toxicity or inflammation, and therefore, the complexes may be suitable for delivery strategies [69,70,71]. Similar results were obtained when the MTT study was performed in CTX-TNA2 cells (Figure 2) for the same time points considered. A cell viability percentage above 80% was reached with all complexes. These results indicated the non-toxicity profile of peptide complexes and their biocompatibility with non-cancer cells. Furthermore, a non-cytotoxic effect was observed for TMZ-loaded and non-loaded systems. This effect demonstrates the potential of TMZ to treat glioma cells without compromising non-cancerous brain cells and the potential of TMZ encapsulation in decreasing side effects [72]. The same results were obtained when performing the MTT assay with fibroblasts and primary lung smooth muscle cells transfected with the different peptide/pDNA complexes (N/P ratio of 1) and support the biocompatibility of the developed systems (Figures S4 and S5, available in the Supplementary Materials). These results are an indication that the complexes are potentially safe and biocompatible delivery systems.

### 3.3. Biocompatibility in Zebrafish Embryos 

The potential toxic effect of the developed peptide complexes on biological systems was evaluated using the zebrafish, *Danio rerio*, as a model organism in a fish embryo toxicity (FET) assay. Zebrafish embryos are a useful, predictive, and well-established model to assess the toxicity displayed by nano-complexes since, as the genomes of these animals and humans are closely related, results can predict potential effects on humans [73,74]. Furthermore, this model is easy to manipulate and economical compared to other animal models [74,75,76]. Since several adults can be maintained in small-scale aquariums, this model system provides several hundreds of eggs in one breeding event. The optical transparency of embryos allows easy visualization of organ morphology and possible malformations, thus avoiding invasive techniques [74,75,76]. Some recent studies have highlighted the importance of this kind of model to test nanoparticle toxicity [73,77,78,79,80,81]. 

Before FET assays, it was crucial to evaluate the stability and physicochemical properties exhibited by the various WRAP5/pDNA complexes after their incubation in zebrafish system water as well as to verify if the complexes kept the pDNA protection capacity. This is a relevant issue to infer as complex stability may affect their performance as delivery vectors, influencing their ability for cellular uptake, payload delivery, and, consequently, therapeutic outcomes. In line with this, the different peptide/pDNA complexes were formulated at N/P ratios of 1, 1×, and 10×, concentrated (to facilitate in vivo experiments, since we had to scale up the quantities used), and incubated for 24, 48, 72, and 96 h in FSW, at 26 °C. The main aim was to closely simulate the experimental conditions of a zebrafish-based in vivo model. The results are presented in the Supplementary Materials, Figures S6–S9 and Table S1. The electrophoretic migration of the complexes (Figures S6 and S7) showed the absence of pDNA bands in the agarose gel after 24, 48, 72, and 96 h of incubation. These results suggested that peptide complexes can remain stable in suspension for at least 96 h and assured pDNA complexation and protection after being subjected to the tested conditions. 

Moreover, the physicochemical properties of the developed complexes were also investigated by DLS after 96 h of incubation. Parameters such as the mean size, PdI, and zeta potential were determined to verify the impact of water composition and temperature on the set of complexes properties. The results are presented in Figures S8 and S9 and Table S1. As observed, the different peptide/pDNA complexes displayed similar properties when formulated at normal concentration or in the condition of a 10 times higher concentration and when incubated in FSW for 96 h. Small differences were noted for PdI values, however, complexes maintained monodisperse distribution in solution. From these data, we concur that the properties of the nano-complexes were not altered by their incubation in zebrafish system water for all time points measured. This fact is particularly relevant since we can confirm the stability and protection of pDNA during zebrafish embryo assays.

**Figure 3:**
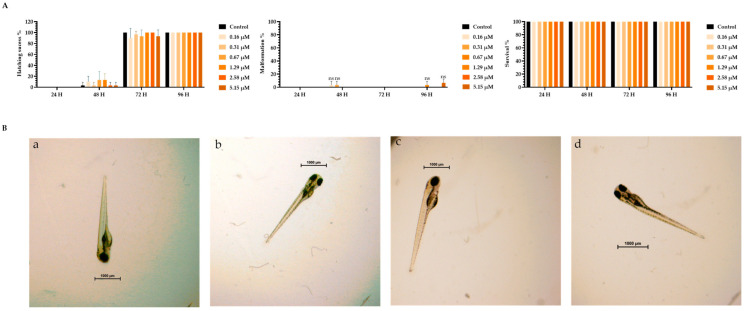
(**A**) Effects of TMZ drug in *Danio rerio* zebrafish embryo–larval hatching success, malformations, and survival over time after exposure to a range of concentrations of 0–5.15 µM of TMZ drug. (**B**) Larval stereomicroscope images of control (**a**), DMSO solvent (**b**), and concentration of 1 mg TMZ drug/L (**c**,**d**) after 96 h of exposure. Scale bar = 1000 μm. ns—non-significant (*p* > 0.5).

**Figure 4:**
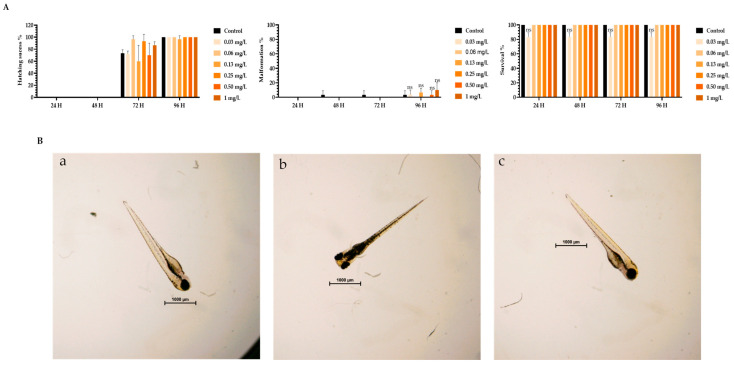
(**A**) Effects of plasmid DNA (pDNA) in *Danio rerio* zebrafish embryo–larval hatching success, malformations, and survival over time after exposure to a range of concentrations of 0–1 mg of pDNA/L. (**B**) Larval stereomicroscope images of control (**a**), and concentration of 1 mg pDNA/L (**b**,**c**) after 96 h of exposure. Scale bar = 1000 μm. ns—non-significant (*p* > 0.5).

**Figure 5:**
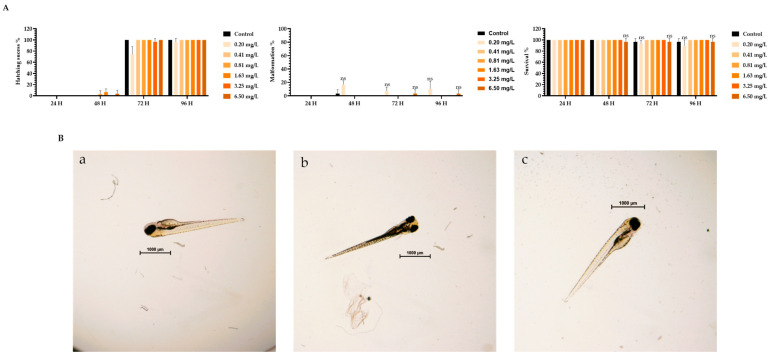
(**A**) Effects of TMZ/WRAP5 peptide in *Danio rerio* zebrafish embryo–larval hatching success, malformations, and survival over time after exposure to a range of concentrations of 0–6.5 mg of TMZ/WRAP5 peptide/L. (**B**) Larval stereomicroscope images of control (**a**), and concentration of 6.5 mg TMZ/WRAP5 peptide/L (**b**,**c**) after 96 h of exposure. Scale bar = 1000 μm. ns—non-significant (*p* > 0.5).

**Figure 6:**
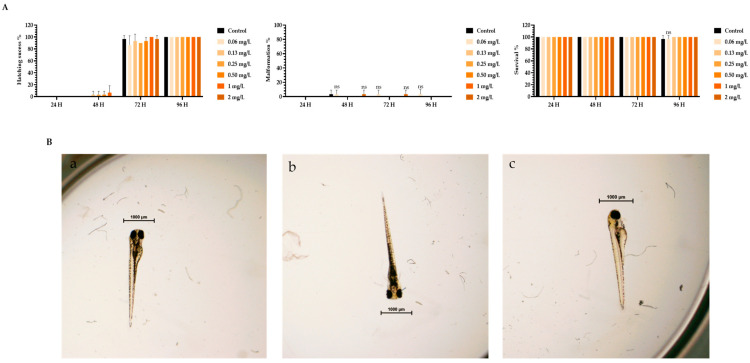
(**A**) Effects of TMZ/Tf-WRAP5 peptide in *Danio rerio* zebrafish embryo–larval hatching success, malformations, and survival in time after exposure to a range of concentrations of 0–2 mg of TMZ/Tf-WRAP5 peptide/L. (**B**) Larval stereomicroscope images of control (**a**), and concentration of 2 mg TMZ/Tf-WRAP5 peptide/L (**b**,**c**) after 96 h of exposure. Scale bar = 1000 μm. ns—non-significant (*p* > 0.5).

**Figure 7:**
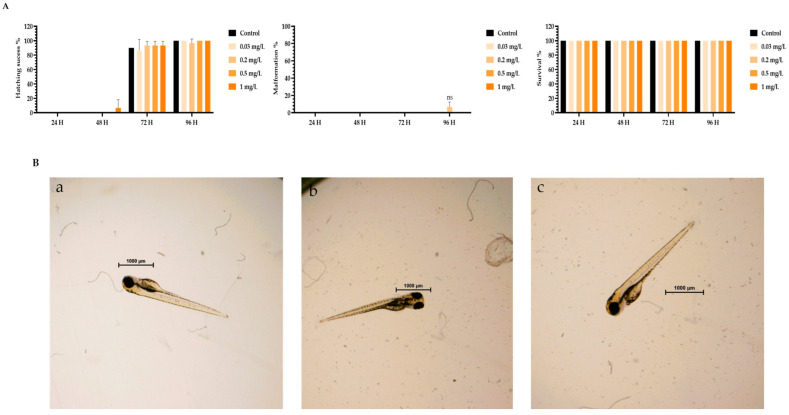
(**A**) Effects of Tf-WRAP5/pDNA at N/P ratio of 1 in *Danio rerio* zebrafish embryo–larval hatching success, malformations, and survival over time after exposure to a range of concentrations of 0–1 mg of pDNA/L. (**B**) Larval stereomicroscope images of control (**a**), and concentration of 2 mg pDNA/L (**b**,**c**) after 96 h of exposure. Scale bar = 1000 μm. ns—non-significant (*p* > 0.5).

**Figure 8:**
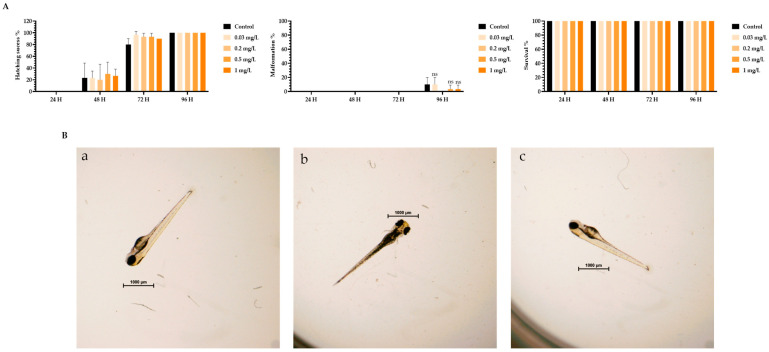
(**A**) Effects of TMZ/Tf-WRAP5/pDNA at N/P ratio of 1 in *Danio rerio* zebrafish embryo–larval hatching success, malformations, and survival over time after exposure to a range of concentrations of 0–1 mg of pDNA/L. (**B**) Larval stereomicroscope images of control (**a**), and concentration of 1 mg pDNA/L (**b**,**c**) after 96 h of exposure. Scale bar = 1000 μm. ns—non-significant (*p* > 0.5).

To assess the toxicity of TMZ drug, pDNA, peptides, and the different peptide/pDNA complexes at an N/P ratio of 1, the zebrafish embryo model was used to determine the complexes’ influence during the first 96 h of embryonic development. The displayed concentration range tested in embryos was designed to include the concentrations of pDNA and peptides used to encapsulate the pDNA 10× formulations. Parameters such as zebrafish embryo–larval hatching success, malformation, and survival were monitored in the toxicity assays. A summary of the obtained results is depicted in Figure 3, Figure 4, Figure 5, Figure 6, Figure 7 and Figure 8.

The toxicity analysis demonstrated that organisms showed a hatching success of over 80% (a process that occurs between 48 and 72 h), non-significant malformations, and survival above 90% after 96 h of exposure in all tested conditions. These assumptions were made by comparing the treatment groups with the negative FSW control since no statistical difference was seen between the solvent control and FSW control (in the case of the TMZ). This outcome shows that the experiments fulfill the criteria of OECD 236 guidelines and are validated. Furthermore, normal hatching occurs between 48 h and 72 h, so the presented results suggest that a normal embryo–larval hatching occurred. Altogether, the results suggest that pDNA complexes had no toxic effects on the zebrafish embryos. The observation of some malformations in control groups (Figure 4, Figure 5, Figure 6 and Figure 8) may be due to natural and random embryo–larval development during the test. 

These results demonstrated the non-toxic effect of the developed nano-complexes on the zebrafish model and supported their biocompatibility. 

Additional studies regarding biomarker analysis and DNA damage on zebrafish after exposure to the complexes are planned as the next step to deeply unravel the embryo–larval toxicity effect. 

### 3.4. WRAP5/pDNA Complexes Are Internalized by Glioblastoma Cells 

**Figure 9:**
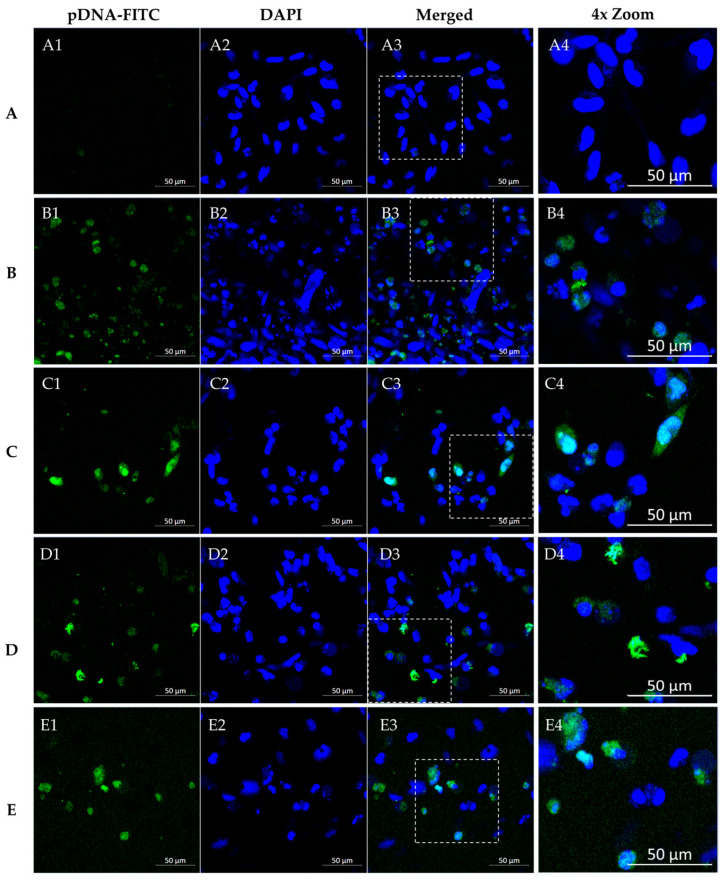
Fluorescence confocal microscopy study. Representative live cell images of SNB19 cells after 4 h of transfection mediated by the different peptide/pDNA complexes developed at N/P ratio of 1 (using 1 µg pDNA). DAPI dye stained the nuclei while FITC labeled pDNA green. Panels (**A1**–**E1**) indicate labeled pDNA-FITC, panels (**A2**–**E2**) show nuclei stained by DAPI, panels (**A3**–**E3**) correspond to merged images, and panels (**A4**–**E4**) present a 4× zoom of the highlighted regions. Non-transfected cells (**A**) and cells transfected with the developed complexes: WRAP5/pDNA-FITC (**B**), Tf-WRAP5/pDNA-FITC (**C**), TMZ/WRAP5/pDNA-FITC (**D**), and TMZ/Tf-WRAP5/pDNA-FITC (**E**). Scale bar = 50 μm.

**Figure 10:**
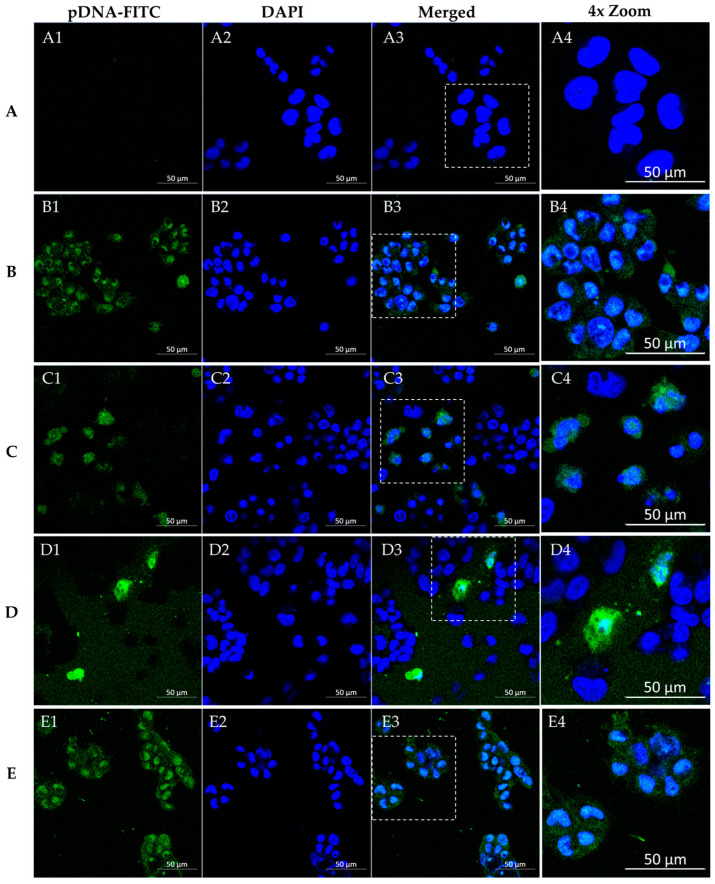
Fluorescence confocal microscopy study. Representative live cell images of U373 cells after 4 h of transfection mediated by the different peptide/pDNA complexes developed at N/P ratio of 1 (using 1 µg pDNA). DAPI dye stained the nuclei while FITC labeled pDNA green. Panels (**A1**–**E1**) indicate labeled pDNA-FITC, panels (**A2**–**E2**) show nuclei stained by DAPI, panels (**A3**–**E3**) correspond to merged images, and panels (**A4**–**E4**) present a 4× zoom of the highlighted regions. Non-transfected cells (**A**) and cells transfected with the developed complexes: WRAP5/pDNA-FITC (**B**), Tf-WRAP5/pDNA-FITC (**C**), TMZ/WRAP5/pDNA-FITC (**D**), and TMZ/Tf-WRAP5/pDNA-FITC (**E**). Scale bar = 50 μm.

A fluorescence confocal microscopy study was conducted in SNB19 and U373 cells to monitor the ability of the conceived WRAP5/pDNA systems to target and internalize into glioma cells. Experiments were performed in live cell mode. As referred to in the experimental section, DAPI was applied to stain the nuclei (blue) and pDNA was stained green by FITC. Microscopy SNB19 cell images are shown in Figure 9. Untreated cells served as a control, as evidenced by the absence of fluorescence (Figure 9(A1–A3)). The next set of images confirmed that the complexes entered the cells, with labeled pDNA-FITC in the cytoplasm and perinuclear space of tumoral cells, but they were also present in the nucleus (merged image). Furthermore, staining was weaker for complexes not bearing the Tf ligand (Figure 9(B1–B3,D1–D3)), indicating a poor capacity of these systems for cellular internalization, and, thus, cellular transfection. On the contrary, when SNB19 cells were transfected by Tf-WRAP5/pDNA-FITC and TMZ/Tf-WRAP5/pDNA-FITC nano-systems, images demonstrated that internalization occurred to a greater extent, with pDNA-FITC located in the nucleus (Figure 9(C1–C3,E1–E3)). Figure 10 presents microscopy images from U373 cells when these cells were transfected with the same peptide/pDNA complexes. As for SNB19 cells, the live cell experiment proved the successful internalization of the delivery systems into the cells (Figure 10(B1–B3,C1–C3, D1–D3,E1–E3)), although some differences between the complexes were observed. As for SNB19 cells, when transfection is performed using complexes without Tf ligand, a less intense fluorescence signal from the stained pDNA is detected (Figure 10(B1–B3,D1–D3)). Moreover, a more efficient cellular uptake can be predicted from images corresponding to transfection with the Tf-WRAP5/pDNA-FITC and TMZ/Tf-WRAP5/pDNA-FITC systems (Figure 10(C1–C3,E1–E3)). The merged images revealed a strong accumulation of pDNA in the nucleus (Figure 10(C3,E3)). Moreover, quantification of the ratio of green fluorescence intensity per nucleus, as shown in the Supplementary Materials—Figure S10—also supported the role of Tf in the cellular uptake and the efficacy of TMZ/Tf-WRAP5/pDNA to be internalized into glioma cells.

These observations on both SNB19 and U373 cells agreed well with a previous similar study performed by our team on U87 cells [43]. Data had shown an efficient internalization of Tf-WRAP5/pDNA-FITC and TMZ/Tf-WRAP5/pDNA-FITC complexes into these cells. Additionally, pDNA successfully reached the nucleus, as merged images show. The functionalization with the Tf ligand at the complexes’ surface made the penetration of peptide complexes into the glioma cells easier via transferrin receptor-mediated endocytosis [43,82,83,84]. This phenomenon of improved cellular uptake related to the functionalization of vectors with Tf has also been described by other authors for the same cells [85,86]. Moreover, a preliminary microscopy study on U87 glioblastoma spheroids showed the internalization of the developed peptide complexes into a 3D glioblastoma model. Making the connection between 2D cell culture and in vivo animal models, spheroidal models of glioblastoma will be further explored, by our team, for a deeper insight into the potential therapeutic effect mediated by the developed WRAP5-based drug/gene complexes. 

### 3.5. p53 Protein Is Expressed in Glioblastoma Cells Transfected with WRAP5/pDNA Complexes

**Figure 11:**
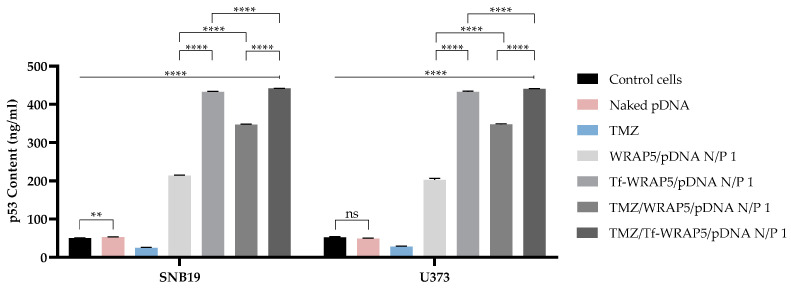
p53 protein levels in SNB19 and U373 cells after 48 h of transfection mediated by the different peptide/pDNA complexes developed at N/P ratio of 1 (using 1 µg pDNA). Data were obtained from six independent measurements (mean ± SD, n = 6) and analyzed by one-way ANOVA, followed by the Bonferroni test. **** *p* < 0.001; ** *p* < 0.021; ns—non-significant (*p* > 0.5).

In p53-based therapies, it is relevant to assess/quantify p53 protein cell expression levels as an indication of effective cellular transfection and, therefore, expected therapeutic outcomes [43]. Quantifying p53 allows us to evaluate the capacity of the formed complexes to efficiently deliver pDNA to glioma cells and promote gene expression. Since we had evidence of peptide/pDNA complexes’ internalization and nucleus pDNA co-localization, p53 expression in glioma cell lines was quantified through an ELISA immunoassay 48 h post-transfection. Untreated cells were considered as controls. Figure 11 summarizes the results obtained for p53 content quantification in SNB19 and U373 cells. The quantification of p53 after transfection of SNB19 cells with the developed complexes revealed that all four complexes were capable of inducing a significant increase in p53 protein content comparatively to non-transfected cells (**** *p* ≤ 0.0001), with the exception of pDNA (naked) that presented similar protein content in relation to control cells (** *p* ≤ 0.01). This underlines the importance of pDNA complexation into the nanocarriers to protect and deliver it and guarantee a successful transfection process for posterior protein translation. Also, the p53 content varied within the complexes used in cellular transfection. Tf-WRAP5/pDNA and TMZ/Tf-WRAP5/pDNA complexes were able to induce p53 production to a higher extent compared to WRAP5/pDNA and TMZ/WRAP5/pDNA (**** *p* ≤ 0.0001). The difference in protein content may be associated with a more efficient cell uptake and internalization and later accumulation of pDNA in the nucleus, with complexes loading the Tf targeting sequence. This may enhance the overall gene expression, resulting in higher protein production. The presence of TMZ in the complexes also appeared to affect produced p53 levels (**** *p* ≤ 0.0001 for WRAP5/pDNA and Tf-WRAP5/pDNA versus TMZ/WRAP5/pDNA and TMZ/Tf-WRAP5/pDNA). It seemed that the conceived TMZ complexes were able to promote the production of higher p53 levels, most probably as TMZ activates the p53 pathway [30,87,88,89]. In U373 cells, the results for p53 content quantification post-transfection with the same peptide/pDNA complexes followed the same tendency as for SNB19 cells. All the complexes were capable of inducing a significant increase in p53 protein cell content compared to non-transfected cells (**** *p* ≤ 0.0001), except naked pDNA which presented a non-significant statistical difference compared to the control cells. Similarly, transfection mediated by complexes containing Tf and TMZ resulted in higher p53 levels when compared with protein content achieved with complexes not bearing the ligand and the drug (**** *p* ≤ 0.0001). These findings agree well with the previous images obtained from the confocal microscopy study, where WRAP5/pDNA complexes bearing the Tf proved to be internalized to a greater extent. The results for both cells lines are in agreement with p53 quantification data of U87 cells also described and discussed in previous work [43]. p53 quantification revealed a successful protein expression post-transfection with the complexes. Once more, transfection mediated by the complexes functionalized with Tf was more efficient at increasing p53 levels than transfection mediated by the systems not bearing the sequence. However, TMZ did not seem to affect protein expression in U87 cells. No statistically significant difference was obtained between complexes with TMZ and the ones where TMZ was absent. 

Another important observed fact was the similarity of p53 protein levels in control cells between SNB19 and U373 and the previously studied U87 cell line [43]. SNB19 and U373 cells expressed mutant-type TP53 while U87 expressed wild-type TP53. According to several studies, in mutated cell lines, p53 protein levels are not affected but the expressed protein is mutated, with the loss or gain of function or mutational effects of dominant-negative type [90,91]. It is also known that this leads to a more aggressive tumor profile since TP53 mutation is associated with tumorigenesis, a worse prognosis, and ineffective responses to treatments [90,92]. The obtained results reinforce the great potential of the developed formulations in supplementing p53 mutated cancer cells and the value of gene therapy approaches toward glioblastoma.

### 3.6. Tf-WRAP5/pDNA and TMZ/Tf-WRAP5/pDNA Complexes Reduce Glioblastoma Cell Viability

**Figure 12:**
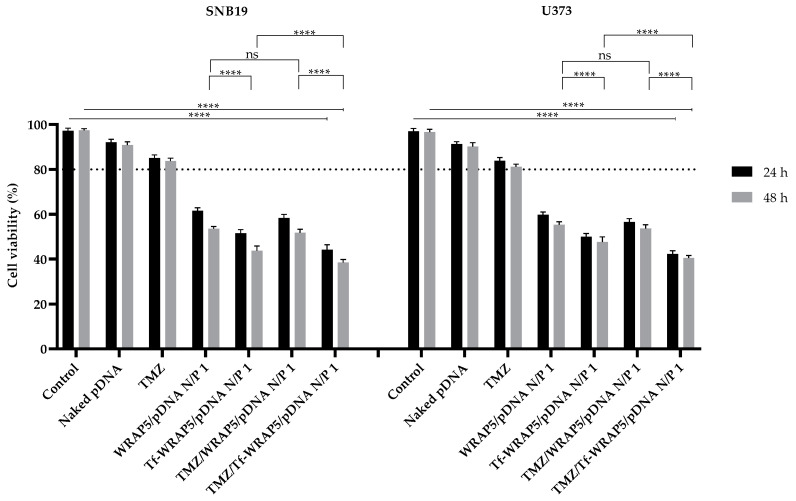
Cellular viability of SNB19 and U373 cells after 24 and 48 h of transfection mediated by the different peptide/pDNA complexes developed at N/P ratio of 1 (using 1 µg pDNA). Cells not transfected were used as a positive control and cells treated with naked pDNA and TMZ drug were used as controls. Data were obtained from six independent measurements (mean ± SD, n = 6) and analyzed by one-way ANOVA, followed by the Bonferroni test. The dotted line represents the limit accepted for a substance to be considered as non-cytotoxic. **** *p* < 0.001; ns—non-significant (*p* > 0.5).

After assessing the p53 content in glioma cells and discussing the effect of developed complexes on p53 expression levels, it was of relevance to investigate how p53 activation influences cancer cell viability. The cytotoxic effect in the two cell lines under study was evaluated at 24 h and 48 h through an MTT colorimetric experiment. Cells not transfected were taken as a positive control. Cells transfected with naked pDNA and TMZ alone were also considered for comparative purposes. Figure 12 displays the cell viability percentages of SNB19 and U373 cells achieved with their transfection with the different complexes. For SNB19 cells, a prominent decrease in viability was found for all complexes (**** *p* ≤ 0.0001) at both time points, but it was more pronounced 48 h post-transfection. A higher decrease in cellular viability was found for cells transfected with Tf-WRAP5/pDNA and TMZ/Tf-WRAP5/pDNA complexes relative to WRAP5/pDNA and TMZ/WRAP5/pDNA (at 48 h, **** *p* ≤ 0.0001). In addition, there was also a significant difference between the incubation of SNB19 cells with Tf-WRAP5/pDNA and TMZ/Tf-WRAP5/pDNA complexes at 48 h (**** *p* ≤ 0.0001). This difference was not significant when comparing the WRAP5/pDNA complexes with TMZ/WRAP5/pDNA (at 48 h, ns). In the end, Tf-containing complexes reduced cell viability the most, particularly TMZ/Tf-WRAP5/pDNA complexes, with a loss of viability of around 62% at 48 h. For U373 cells, the observations were very similar, with the developed complexes demonstrating their ability to reduce cell viability efficiently compared to control cells (**** *p* ≤ 0.0001). Comparatively to WRAP5/pDNA and TMZ/WRAP5/pDNA complexes, Tf-WRAP5/pDNA and TMZ/Tf-WRAP5/pDNA induced a higher loss of cell viability (at 48 h, **** *p* ≤ 0.0001). The comparison between Tf-WRAP5/pDNA and TMZ/Tf-WRAP5/pDNA systems was statistically different at 48 h (**** *p* ≤ 0.0001). When comparing WRAP5/pDNA with TMZ/WRAP5/pDNA, the difference was not statistically significant. TMZ/Tf-WRAP5/pDNA was revealed to be the most effective in decreasing the viability of U373 cells, with a loss of approximately 60% of cell viability after 48 h. Improved cell targeting, uptake, internalization, and consequent p53 protein expression resulted in higher cell viability loss for SNB19 and U373 cell transfection mediated by Tf-WRAP5/pDNA and TMZ/Tf-WRAP5/pDNA. Data from U87 cells presented in the previous study [43] showed some differences from the profile found here for SNB19 and U373 cells. For all complexes, the viability decreased as incubation time increases (at 48 h, **** *p* ≤ 0.0001). Tf sequence also proved to influence cellular viability as there were statistically significant differences between WRAP5/pDNA and Tf-WRAP5/pDNA (at 48 h, ** *p* ≤ 0.01) and TMZ/WRAP5/pDNA and TMZ/Tf-WRAP5/pDNA (at 48 h, **** *p* ≤ 0.0001). On the other hand, TMZ seemed to have an accentuated effect in enhancing the loss of cell viability in this wtTP53 glioma cell line (at 48 h, ** *p* ≤ 0.01 for WRAP5/pDNA versus TMZ/WRAP5/pDNA complexes and **** *p* ≤ 0.0001 for Tf-WRAP5/pDNA versus TMZ/Tf-WRAP5/pDNA). A loss of 60% was also found for the transfection with the latter nanos-systems.

Restoring p53 protein function and its pathway has been a successful approach for glioblastoma treatment. Additionally, restoring normal p53 levels in p53 mutants can sensitize cancer cells to TMZ action [30,31,32,33,34]. We may speculate that in SNB19 and U373 cells, both p53 mutants, the cell transfection with developed peptide/pDNA complexes increased drug response and apoptosis induction by TMZ. p53 upregulation may decrease TMZ resistance by suppressing O-6-methylguanine-DNA methyltransferase (MGMT) expression, a DNA enzyme playing a role in the repair process of TMZ-induced DNA damage [32,34]. Furthermore, the p53 tumor suppressor protein has been shown to regulate the expression of some DNA methyltransferases (DNMTs) responsible for methylating DNA groups [35,36]. p53 also induces apoptotic TMZ-independent apoptosis and leads to cell cycle G1 arrest and participates in the activation of several transcriptional genes involved in DNA repair pathways and senescence [37]. These facts may explain the data on cytotoxic effect obtained for TMZ/Tf-WRAP5/pDNA complexes after 48 h of transfection. U87 glioma cells are relatively sensitive to TMZ action, as these cells do not express MGMT [89,93]. This can support the data shown for complexes carrying TMZ, i.e., these complexes gave rise to the most pronounced decrease in cellular viability. 

Furthermore, a comparison of MTT data from SNB19 and U373 cells to those obtained for non-cancer cells discussed earlier in this report (Figure 1 and Figure 2) clearly demonstrates the high specificity of peptide/pDNA complexes to exert a toxic/therapeutic action on glioma cells while protecting healthy cells from toxicity. Altogether, these results on cellular viability evidenced the role of the conceived complexes in inhibiting glioblastoma cell growth/proliferation. 

### 3.7. Tf-WRAP5/pDNA and TMZ/Tf-WRAP5/pDNA Complexes Enhance Apoptosis in Glioblastoma Cells

Apoptosis is a type of programmed cell death, executed as a response to internal (intrinsic or mitochondrial) or external (extrinsic or death receptors pathway) stimuli to safeguard cell function and survival, involving cysteinyl-aspartate-specific proteases [19,94]. The two main apoptosis pathways occur by the action of effector molecules of cell death, the set of initiators and executioner caspases. Caspase-3 is an execution caspase whose activation occurs at the end of both extrinsic and intrinsic pathways and caspase-9 is an initiator caspase of the intrinsic pathway [19]. The B-cell lymphoma 2 (Bcl-2) protein family controls the activation of this latter pathway. In response to various apoptotic stimuli, only BH-3 proteins can be activated, which in turn activate Bcl-2-associated X protein (Bax) and Bak. Bax and Bak accumulate at the mitochondrial outer membrane, promoting its permeabilization, a key step in apoptosis [19,95].

**Figure 13:**
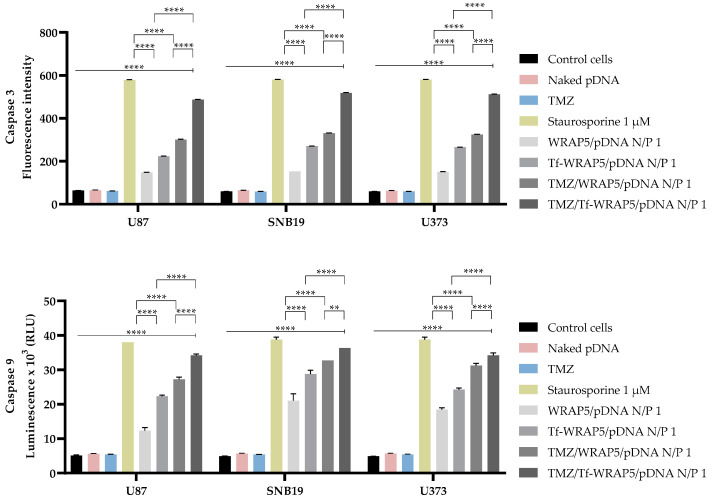
Caspase-3 (above) and caspase-9 (below) activity in U87, SNB19, and U373 cells after transfection (48 h) mediated by the different peptide/pDNA complexes developed at N/P ratio of 1 (using 1 µg pDNA). Positive control refers to the study with staurosporine (1 μM), while negative control considers untreated cells. Data were obtained from three independent measurements (mean ± SD, n = 3) and analyzed by one-way ANOVA, followed by the Bonferroni test. **** *p* < 0.001; ** *p* < 0.021.

Caspase-3 and caspase-9 activities were assessed after 48 h of cellular transfection to assess the induction of apoptosis mediated by the nano-systems and determine which pathway is activated. In previous work, our research group reported the first results regarding the dual delivery of developed systems to U87 cells, as has been discussed [43]. Figure 13 includes new and complementary results regarding the U87 glioma cell line and summarizes the obtained results for the SNB19 and U373 cell lines studied in this manuscript. In U87 cells, all the complexes led to the activation of both caspase activities (**** *p* ≤ 0.0001). A remarkable difference was found between WRAP5/pDNA and TMZ/WRAP5/pDNA and Tf-WRAP5/pDNA and TMZ/Tf-WRAP5/pDNA complexes (**** *p* ≤ 0.0001). Furthermore, an accentuated difference was also noted between WRAP5/pDNA and Tf-WRAP5/pDNA and the complexes having TMZ, TMZ/WRAP5/pDNA, and TMZ/Tf-WRAP5/pDNA (**** *p* ≤ 0.0001). TMZ complexes were the ones inducing higher caspase activation. In SNB19 and U373 cells, transfection with the complexes also resulting in the activation of both caspases (**** *p* ≤ 0.0001). A statistical difference between complexes with and without Tf ligand (**** *p* ≤ 0.0001) and with and without TMZ (**** *p* ≤ 0.0001, ** *p* ≤ 0.01 in SNB19 cells for caspase-9 activity) was also noticed. Again, TMZ complexes were the ones inducing a greater extent of caspase activation.

Since caspase-9 is activated in all glioma cell lines, it is thus a strong indication that the intrinsic route is activated post-transfection. For U87 cells, which are more sensitive to the TMZ action as these cells are MGMT negative, TMZ seemed to have a higher anticancer action compared to its action in SNB19 and U373 cells. This corroborates well with the already observed effect of TMZ-bearing complexes decreasing U87 cell viability the most [43]. The capacity of TMZ to induce caspase activity and apoptosis in U87 cells has been described and discussed in the literature [96,97]. As SNB19 and U373 cells are resistant to TMZ action and TMZ-loaded complexes were not the ones decreasing SNB19 and U373 cellular viability the most and given that these complexes were able to promote activation of caspases, most probably, a senescence phenomenon took place in these cells due to TMZ treatment. According to published studies, TMZ drug can induce apoptosis, but it also activates survival pathways such as senescence [98,99]. Considering that MGMT protein may exert an important role in TMZ resistance, it would be advantageous to test MGMT expression in the investigated cell lines [34,100]. Experimental work on this theme is ongoing and, hopefully, will be reported soon to add some clues contributing to solving the complexity of this matter.

Moreover, Bax protein expression levels in glioma cells post-transfection with the developed peptide/pDNA nano-systems were quantitatively determined for U87, SNB19, and U373 as presented in the SM, Figure S11. For all cell lines, all peptide complexes were able to activate Bax (**** *p* ≤ 0.0001 versus the negative control). A similar pattern to the one observed for caspases-3 and -9 activations was found also for the Bax activation, i.e., a significant difference was found between WRAP5/pDNA and TMZ/WRAP5/pDNA (**** *p* ≤ 0.0001) and Tf-WRAP5/pDNA and TMZ/Tf-WRAP5/pDNA (**** *p* ≤ 0.0001) and between WRAP5/pDNA and Tf-WRAP5/pDNA (**** *p* ≤ 0.0001) and TMZ/WRAP5/pDNA and TMZ/Tf-WRAP5/pDNA complexes (**** *p* ≤ 0.0001). Again, TMZ complexes were the ones inducing a greater extent of Bax protein production. 

Bax expression in cancer cells supports the evidence of induced apoptosis by the nano-complexes via the intrinsic pathway.

## 4. Conclusions

Glioblastoma is an aggressive type of tumor that affects the brain tissue. Conventional treatment includes surgery followed by radiotherapy and chemotherapy. However, the main challenges, like the localization of the tumor, ineffective medication distribution across the tumor, and drug resistance, greatly impair the overall patients’ survival and it becomes imperative to seek new therapy approaches. In the gene therapy context, non-viral vectors emerge as innovative platforms to improve glioma treatment efficacy. Along with this, developing a nano-system for combined drug/gene delivery, targeted specifically to glioma cells, is considered a major asset. To fulfill this, our team formulated complexes based on the WRAP5 peptide, a cationic and amphipathic CPP, and a plasmid encoding for the tumor suppressor p53 gene. Therefore, copies of the p53 gene can be delivered to target cells to restore p53 gene levels, re-establishing protein content and thus normal tumor suppressor function [101,102,103]. To ensure specific cell targeting, the Tf ligand was coupled to the peptide, so the plasmid could target the brain tumor. Inside the cell, we take advantage of normal cellular mechanisms, and the plasmid is transcribed in the nucleus and translated into the cytoplasm to produce an active wild-type p53 protein. This approach is based on supplementing defective cells to correct the abnormal p53 levels that contribute to tumor progression. TMZ, a typical drug applied in brain tumors, was also incorporated into the complexes to enhance the therapeutic effect. TMZ/p53 gene peptide complexes formulated at an N/P ratio of 1 were selected due to their suitable properties for in vitro studies. The set of developed WRAP5/pDNA complexes was revealed to be non-toxic to non-cancerous brain cells and to a zebrafish embryo model within the tested conditions. Confocal microscopy experiments evidenced the great capacity of the conceived drug/gene systems for glioma cell internalization, with pDNA localization in the nucleus and perinuclear space. In addition, efficient p53 gene delivery is conducive to p53 protein production. In line with this, a cytotoxic effect was observed in SNB19 and U373 glioma cells, especially 48 h after the transfection with TMZ/Tf-WRAP5/pDNA complexes. Moreover, caspases-3 and -9 activity quantification, as well as Bax protein quantification, revealed the capacity of the nano-complexes to promote cancer cells apoptosis, via the intrinsic pathway. 

Overall, we report on a novel co-delivery platform displaying glioma-targeting ability and high capacity for cellular transfection, p53 expression, and apoptosis induction.

Future studies will be aimed at evaluating the therapeutic effect of the developed peptide-based complexes in 3D cell culture models and in vivo research using a zebrafish model.

## Figures and Tables

**Table 1:** The set of properties, peptide-drug-loading efficiency (DLE), mean size, polydispersity index (PdI), average zeta potential determined by dynamic light scattering, and pDNA complexation capacity (CC) displayed by WRAP5/pDNA, Tf-WRAP5/pDNA, TMZ/WRAP5/pDNA, and TMZ/Tf-WRAP5/pDNA complexes developed at N/P ratio of 1 (using 1 µg pDNA). Data were obtained from three independent measurements (mean ± SD, n = 3). Adapted from Reference [43].

System	Drug Loading Efficiency (DLE) (%)	Size (nm)	PdI	Zeta Potential (mV)	CC (%)
WRAP5/pDNA	-	271.7 ± 5.00	0.300 ± 0.03	+4.03 ± 0.11	89.67 ± 4.04
Tf-WRAP5/pDNA	-	179.9 ± 4.00	0.410 ± 0.04	+12.55 ± 0.42	92.56 ± 1.81
TMZ/WRAP5/pDNA	60.1 ± 4.8	232.7 ± 6.00	0.327 ± 0.03	+4.43 ± 0.05	94.33 ± 0.58
TMZ/Tf-WRAP5/pDNA	66.4 ± 8.3	182.9 ± 4.00	0.396 ± 0.04	+11.94 ± 0.29	89.56 ± 2.19

## Data Availability

The data presented in this study are available in this article and Supplementary Materials.

## Supplementary Materials

The following supporting information can be downloaded at: https://www.mdpi.com/article/10.3390/pharmaceutics16060781/s1, Scheme S1: TMZ hydrolysis in water to form the 5-aminoimidazole-4-carboxamide (AIC) and the intermediate methyl diazonium ion products; Figure S1: Electrophoretic analysis of the pDNA protection capacity displayed by the complexes after their incubation with 10% fetal bovine serum (FBS); Figure S2: In vitro hemolysis test using red blood cells (RBCs) treated with WRAP5/pDNA, Tf-WRAP5/pDNA, TMZ/WRAP5/pDNA, and TMZ/Tf-WRAP5/pDNA complexes developed at N/P ratio of 1 (using 1 µg pDNA); Figure S3: IL-6 and IL-1β cytokine concentration after transfection mediated by the different peptide/pDNA complexes developed at N/P ratio of 1 (using 1 µg pDNA). Figure S4: Cellular viability of fibroblasts after 24 h, 48 h, and 72 h of transfection mediated by the different peptide/pDNA complexes developed at N/P ratio of 1 (using 1 µg pDNA); Figure S5: Cellular viability of primary lung smooth muscle cells after 24 h, 48 h, and 72 h of transfection mediated by the different peptide/pDNA complexes developed at N/P ratio of 1 (using 1 µg pDNA); Figure S6: Electrophoretic analysis of WRAP5/pDNA, Tf-WRAP5/pDNA, TMZ/WRAP5/pDNA, and TMZ/Tf-WRAP5/pDNA complexes developed at N/P ratio of 1 (using 1 µg pDNA) after 24 h and 48 h of incubation in fish system water (FSW) at 26 °C; Figure S7: Electrophoretic analysis of WRAP5/pDNA, Tf-WRAP5/pDNA, TMZ/WRAP5/pDNA, and TMZ/Tf-WRAP5/pDNA complexes developed at N/P ratio of 1 (using 1 µg pDNA) after 72 h and 96 h of incubation in fish system water (FSW) at 26 °C; Figure S8: Mean size of WRAP5/pDNA, Tf-WRAP5/pDNA, TMZ/WRAP5/pDNA, and TMZ/Tf-WRAP5/pDNA complexes developed at N/P ratio of 1 (using 1 µg pDNA) and re-suspended in fish system water (FSW) after 0 (controls) and 96 h of incubation; Figure S9: Average zeta potential of WRAP5/pDNA, Tf-WRAP5/pDNA, TMZ/WRAP5/pDNA, and TMZ/Tf-WRAP5/pDNA complexes developed at N/P ratio of 1 (using 1 µg pDNA) and re-suspended in fish system water (FSW); Figure S10: Green fluorescence intensity per nucleus after 4 h of transfection mediated by the WRAP5/pDNA and TMZ/Tf-WRAP5/pDNA complexes developed at N/P ratio of 1; Figure S11: Bax protein levels in SNB19, U373, and U87 cells 48 h post-transfection mediated by the different peptide/pDNA complexes developed at N/P ratio of 1 (using 1 µg pDNA); Table S1: Polydispersity index of WRAP5/pDNA, Tf-WRAP5/pDNA, TMZ/WRAP5/pDNA, and TMZ/Tf-WRAP5/pDNA complexes developed at N/P ratio of 1 (using 1 µg pDNA) and re-suspended in fish system water (FSW) after 0 (control) and 96 h of incubation.
