# Supplementary material for: Evidence That a Peptide-Drug/p53 Gene Complex Promotes Cognate Gene Expression and Inhibits the Viability of Glioblastoma Cells
Source: Pharmaceutics. 2024 Jun 8;16(6):781. doi: 10.3390/pharmaceutics16060781 (PMC11207567; doi:10.3390/pharmaceutics16060781)
Supplement: Supplementary file 1 [file pharmaceutics-16-00781-s001.zip › pharmaceutics-2984474-supplementary.pdf]

pharmaceutics

Supplementary Material

Evidence That a Peptide-Drug/p53 Gene Complex Promotes Cognate Gene Expression and Inhibits the Viability of Glioblastoma Cells

Ana Neves, Tânia Albuquerque, Rúben Faria, Cecília R. A. Santos, Eric Vivès, Prisca Boisguérin, Diana Carneiro, Daniel F. Bruno, Maria D. Pavlaki, Susana Loureiro, Ângela Sousa and Diana Costa

1. Materials and Methods

1.1. Materials

Agarose and GreenSafe premium were obtained from NZYTech (Lisbon, Portugal) and GRS Universal Ladder from GRiSP (Porto, Portugal). Synthetic sea salt for *Danio rerio* (zebrafish) embryo growth was purchased from Instant Ocean (Spectrum Brands, USA).

Primary Dermal Fibroblast cells (normal, Human adult, HDFa) and Primary Lung Smooth Muscle Cells, normal, Human (PCS-130-010) are from ATCC.

1.2. Methods

1.2.1. pDNA protection assay

WRAP5/pDNA, Tf-WRAP5/pDNA, TMZ/WRAP5/pDNA, and TMZ/Tf-WRAP5/pDNA complexes at N/P ratio of 1 were incubated for 24, 48, 72, and 96 h with 800 µL of filtered *Danio rerio* (zebrafish) embryo salty water (Instant Ocean Synthetic Sea Salt, Spectrum Brands, USA) with a salinity of 0.35, at 26°C. pDNA decomplexation from the complexes was verified in a 1% agarose gel by electrophoresis.

WRAP5/pDNA, Tf-WRAP5/pDNA, TMZ/WRAP5/pDNA and TMZ/Tf-WRAP5/pDNA complexes, all prepared at N/P ratio of 1, were incubated with 10% FBS to perform a stability assay. Complexes were incubated for 6 h with 50 µl of 10% FBS (in PBS, pH 7.4). The release and pDNA degradation were monitored by 1% agarose gel electrophoresis.

To perform the hemolysis assay, 2 mL of fresh blood were collected from rats to a heparinized tube containing EDTA disodium salt and centrifugated at 3000 rpm for 15 min at 4°C to isolate red blood cells (RBCs). Then supernatant was discarded, and RBCs washed with 0.85% w/v NaCl solution until normal saline solution is clear. A 3-5% RBCs suspension was further prepared in PBS pH 7.4 and 900 µl incubated with 100 µl of the different complexes at N/P 1 resuspended in PBS pH 7.4, for 1 h at 37°C. PBS pH 7.4 and Triton-X 100 (1% solution) were used as negative and positive control, respectively. Samples were centrifugated at 8000 rpm for 20 min and the supernatant absorbance read at 576 nm in a UV-vis spectrophotometer. The hemolysis percentage was calculated by using the equation:

$$\text{\% Hemolysis} = \frac{\text{Abs.}_{\text{Sample}} - \text{Abs.}_{\text{Negative control}}}{\text{Abs.}_{\text{Positive control}} - \text{Abs.}_{\text{Negative control}}} \text{ x } 100 \quad (1)$$

1.2.2. Agarose gel electrophoresis

The 1% (w/v) agarose gel was prepared in 100 mL 1x TAE buffer (40 mM Tris base, 20 mM acetic acid, 1 mM EDTA, at pH 8.0) with 1.2 µL of GreenSafe (diluted 1:20 in ultrapure grade water). Electrophoresis ran for 30 min at 150 V and the gel was visualized

with the Uvitec Cambridge Transilluminator (UVItec Limited, Cambridge, United Kingdom).

1.2.3. Characterization of complexes

The properties of peptide/pDNA complexes, such as the mean size, polydispersity index (PdI), and zeta potential, were determined by Dynamic Light Scattering (DLS) using a Zetasizer Nano ZS device (Malvern Instruments, UK). To analyze the mentioned properties, the pellet was suspended in 800 µL of filtered Fish System Water (FSW). The obtained data was analyzed using Malvern Zetasizer software v 6.34.

1.2.4. Cell culture

Fibroblast cells were kept grown in 25 cm^3 T-flasks in Dulbecco's Modified Eagle´s Medium (DMEM)/Hams F-12 Nutrient Mixture (DMEM/F-12) with L-glutamine and 10% heat-inactivated FBS and 1% (v/v) of a mixture of antibiotics composed of penicillin (100 µg/mL) and streptomycin (100 µg/mL). Primary lung smooth muscle cells were maintained in vascular cell basal medium (ATCC, PCS-100-030) supplemented with 5% heat-inactivated FBS, 5% L-glutamine, 0.5 mL penicillin-streptomycin-amphotericin B solution (penicillin 10 units/mL, streptomycin 10 µg/mL and amphotericin B 25 ng/mL), 5 ng/mL of basic-fibroblasts growth factor (b-FGF) and 5 ng/ml epidermal growth factor (EGF), 50 µg/mL of ascorbic acid and 10 ng/mL of insulin. Murine immature dendritic JAWS II cells (ATCC® CRL-11904™) were kept grown in MEM-α medium supplemented with GM-CSF (5 ng/mL) and 10% (v/v) heat-inactivated FBS and 1% (v/v) of the mixture of antibiotics.

1.2.5. Pro-inflammatory IL-6 and IL-1β cytokines *in vitro* production

JAWS II dendritic cells were plated in 96-well plates (10^4 cells/ well) and grown at 37°C and 5% CO_2. On the day of the transfection, 0.1 µg of pDNA was pipetted to each well and left for 24 h. The levels of IL-6 and IL-1β were determined by using commercially ELISA kits, reference number KHC0061 for IL-6 and KHC0011 for IL-1β and following the protocols instructions provided by the manufacturer. The absorbance of the colored products was measured at 450 nm. The intensity of this product is proportional to the IL-6 and IL-1β concentrations. The concentration of each protein was determined from the standard curve.

1.2.6. Cytotoxicity assessment

The cytotoxicity profile of the nano-systems was assessed on fibroblasts and primary lung smooth muscle cells using the MTT assay as described in the manuscript.

2. Results

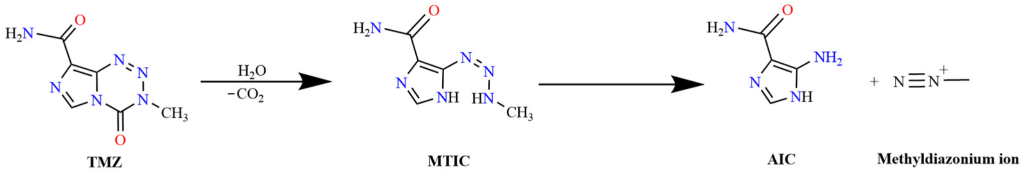

Scheme S1. TMZ hydrolysis in water to form the 5-aminoimidazole-4-carboxamide (AIC) and the intermediate methyl diazonium ion products.

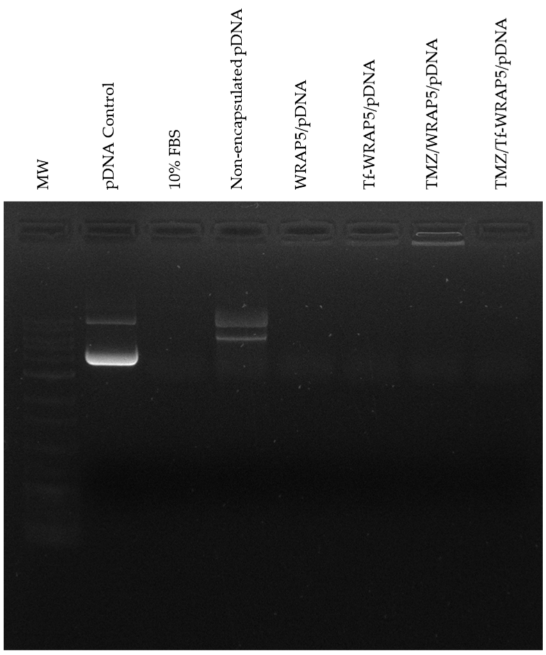

Figure S1. Electrophoretic analysis of the pDNA protection capacity displayed by the complexes after their incubation with 10% fetal serum bovine (FBS). Lane 1: DNA molecular weight marker; Lane 2: pDNA control (1 µg pDNA); lane 3: non-encapsulated pDNA (1 µg pDNA); lane 4: WRAP5/pDNA N/P 1; lane 5: Tf-WRAP5/pDNA N/P 1; lane 6: TMZ/WRAP5/pDNA N/P 1; lane 7: TMZ/Tf-WRAP5/pDNA N/P 1 (using 1 µg pDNA).

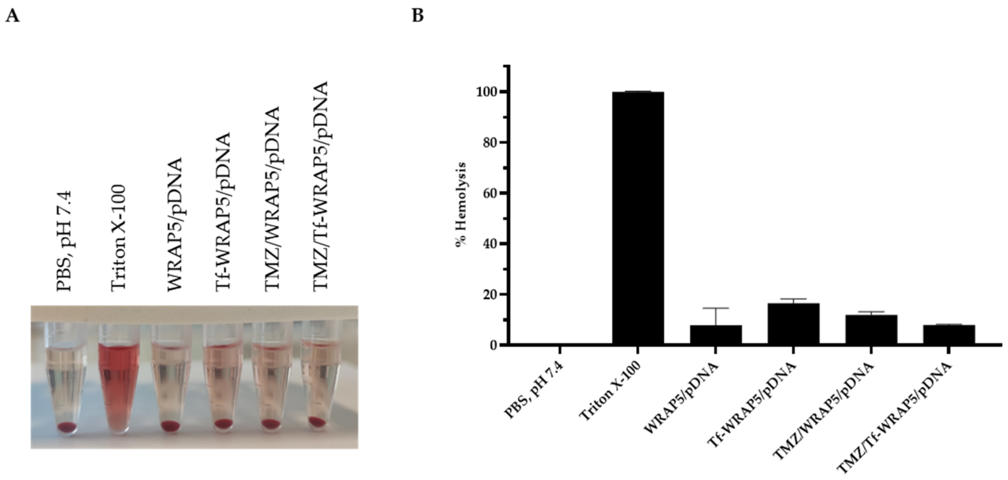

Figure S2. In vitro hemolysis test using red blood cells (RBCs) treated with WRAP5/pDNA, Tf-WRAP5/pDNA, TMZ/WRAP5/pDNA and TMZ/Tf-WRAP5/pDNA complexes developed at N/P

ratio of 1 (using 1 µg pDNA) (A). Percentage of hemolysis (B). Data are presented as mean ± SD (n = 3).

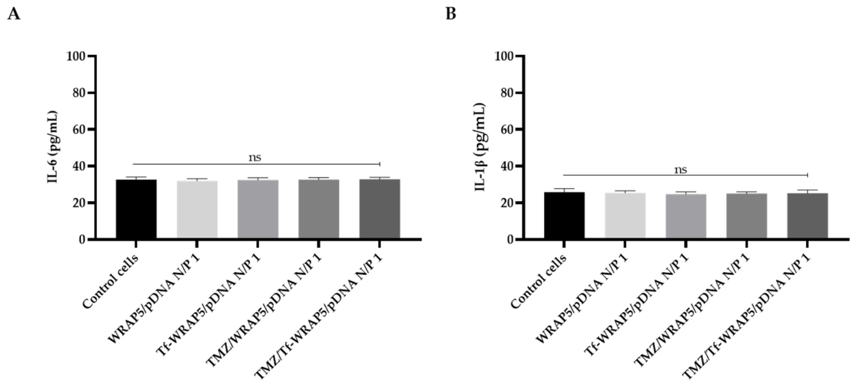

Figure S3. IL-6 (A) and IL-1β (B) cytokine concentration (pg/mL) after transfection mediated by the different peptide/pDNA complexes developed at N/P ratio of 1 (using 1 µg pDNA). Cells not transfected were used as control. Data was obtained from six measurements (mean ± SD, n = 6) and analysed by one-way ANOVA, followed by the Bonferroni test. ns – nonsignificant (p>0.5).

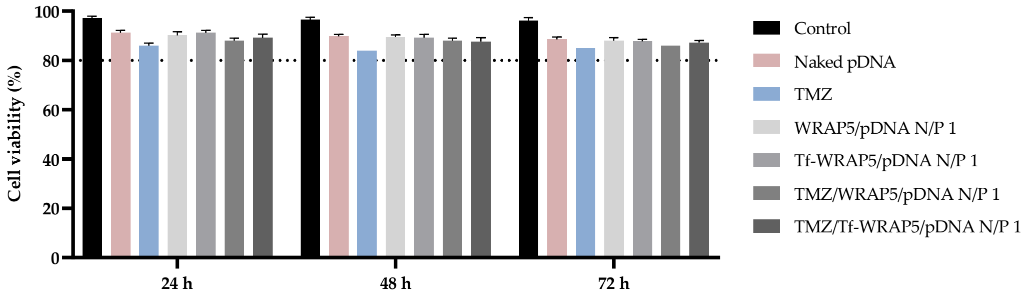

Figure S4. Cellular viability of fibroblasts after 24 h, 48 h, and 72 h of transfection mediated by the different peptide/pDNA complexes developed at N/P ratio of 1 (using 1 µg pDNA). Cells not transfected were used as a positive control and cells treated with naked pDNA and TMZ drug were used as controls. Data was obtained from six independent measurements (mean ± SD, n = 6).

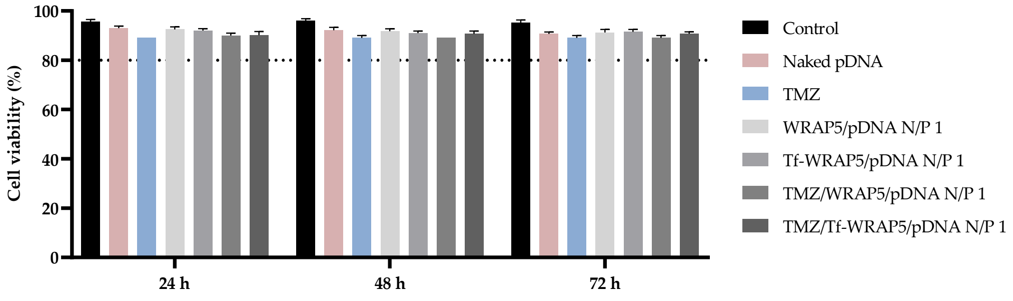

Figure S5. Cellular viability of primary lung smooth muscle cells after 24 h, 48 h, and 72 h of transfection mediated by the different peptide/pDNA complexes developed at N/P ratio of 1 (using 1 µg pDNA). Cells not transfected were used as a positive control and cells treated with naked pDNA and TMZ drug were used as controls. Data was obtained from six independent measurements (mean ± SD, n = 6).

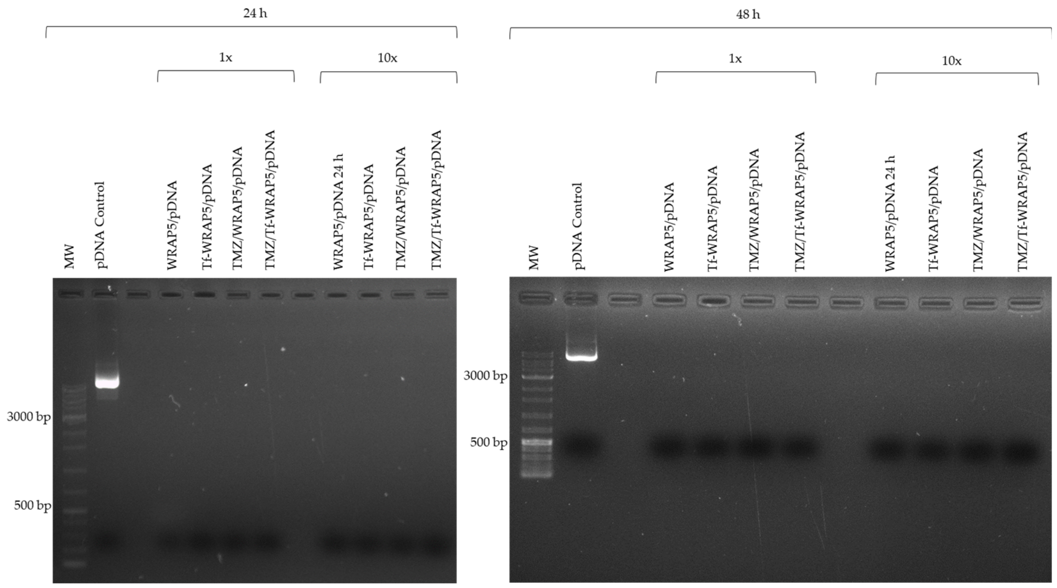

Figure S6. Electrophoretic analysis of WRAP5/pDNA, Tf-WRAP5/pDNA, TMZ/WRAP5/pDNA, and TMZ/Tf-WRAP5/pDNA complexes developed at N/P ratio of 1 (using 1 µg pDNA) after 24 h and 48 h of incubation in Fish System Water (FSW) at 26°C. Lane 1: DNA molecular weight (MW); Lane 2: pDNA control (1 µg pDNA).

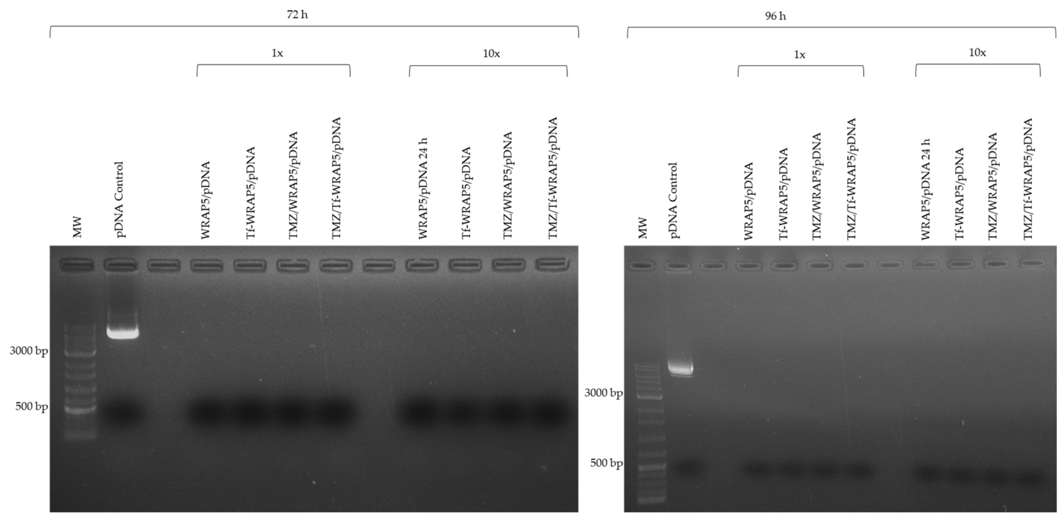

Figure S7. Electrophoretic analysis of WRAP5/pDNA, Tf-WRAP5/pDNA, TMZ/WRAP5/pDNA and TMZ/Tf-WRAP5/pDNA complexes developed at N/P ratio of 1 (using 1 µg pDNA) after 72 h and 96 h of incubation in Fish System Water (FSW) at 26°C. Lane 1: DNA molecular weight (MW); Lane 2: pDNA control.

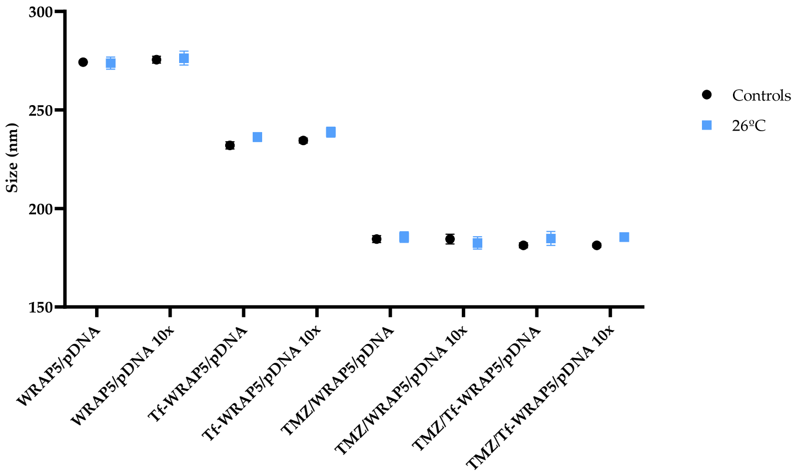

Figure S8. Mean size of WRAP5/pDNA, Tf-WRAP5/pDNA, TMZ/WRAP5/pDNA, and TMZ/Tf-WRAP5/pDNA complexes developed at N/P ratio of 1 (using 1 µg pDNA) and resuspended in Fish System Water (FSW) after 0 (controls) and 96 h of incubation. The values were calculated with the data obtained from four independent measurements (mean ± SD, n = 4).

Table *S*1: Polydispersity index of WRAP5/pDNA, Tf-WRAP5/pDNA, TMZ/WRAP5/pDNA, and TMZ/Tf-WRAP5/pDNA complexes developed at N/P ratio of 1 (using 1 µg pDNA) and resuspended in Fish System Water (FSW) after 0 (control) and 96 h of incubation. The values were calculated with the data obtained from four independent measurements (mean ± SD, n = 4).

**Concentration**	**System**	**Control**	**96 h**
		**PdI**	
1x	WRAP5/pDNA	0.309 ± 0.01	0.332 ± 0.03
	Tf-WRAP5/pDNA	0.264 ± 0.04	0.408 ± 0.04
	TMZ/WRAP5/pDNA	0.393 ± 0.02	0.415 ± 0.02
	TMZ/Tf-WRAP5/pDNA	0.315 ± 0.02	0.316 ± 0.04
10x	WRAP5/pDNA	0.286 ± 0.04	0.297 ± 0.05
	Tf-WRAP5/pDNA	0.398 ± 0.01	0.275 ± 0.03
	TMZ/WRAP5/pDNA	0.386 ± 0.01	0.282 ± 0.04
	TMZ/Tf-WRAP5/pDNA	0.389 ± 0.01	0.276 ± 0.04

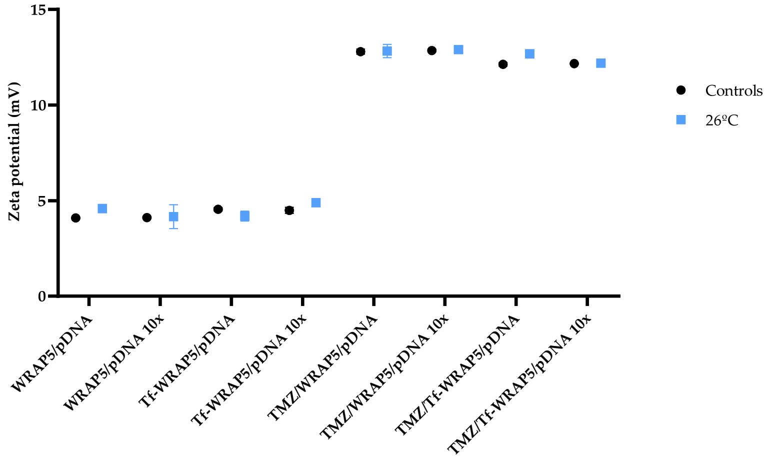

Figure S9. Average zeta potential of WRAP5/pDNA, Tf-WRAP5/pDNA, TMZ/WRAP5/pDNA, and TMZ/Tf-WRAP5/pDNA complexes developed at N/P ratio of 1 (using 1 µg pDNA) and resuspended in Fish System Water (FSW). The values were calculated with the data obtained from four independent measurements (mean ± SD, n = 4).

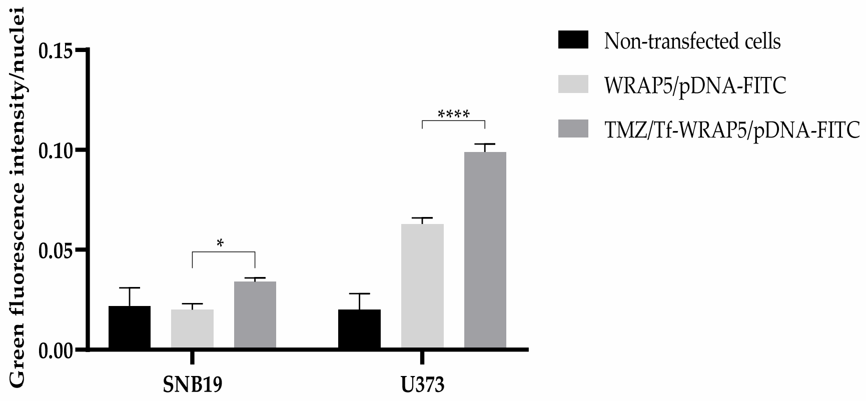

Figure S10. Green fluorescence intensity per nuclei after 4 h of transfection mediated by the WRAP5/pDNA and TMZ/Tf-WRAP5/pDNA complexes developed at N/P ratio of 1 (using 1 µg pDNA). Data was obtained from three independent measurements (mean ± SD, n = 3), and analysed by two-way ANOVA, followed by the Bonferroni test. **** $p<0.001$; * $p<0.332$.

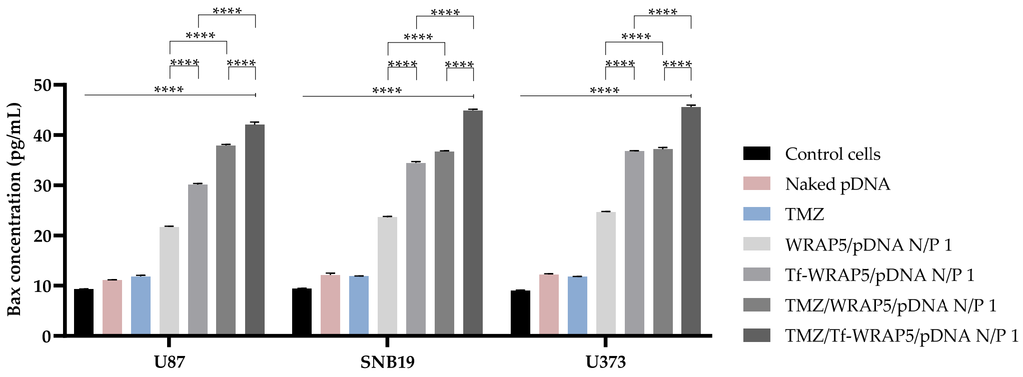

Figure S11. Bax protein levels in U87, SNB19 and U373 cells 48 h post-transfection mediated by the different peptide/pDNA complexes developed at N/P ratio of 1 (using 1 μg pDNA). Untreated cells were considered as a negative control. Data was obtained from three independent measurements (mean ± SD, n = 3) and analysed by one-way ANOVA, followed by the Bonferroni test. **** $p<0.001$.
